# [*PSI^+^*] Maintenance Is Dependent on the Composition, Not Primary Sequence, of the Oligopeptide Repeat Domain

**DOI:** 10.1371/journal.pone.0021953

**Published:** 2011-07-08

**Authors:** James A. Toombs, Nathan M. Liss, Kacy R. Cobble, Zobaida Ben-Musa, Eric D. Ross

**Affiliations:** 1 Department of Biochemistry and Molecular Biology, Colorado State University, Fort Collins, Colorado, United States of America; 2 Graduate Program in Cell and Molecular Biology, Colorado State University, Fort Collins, Colorado, United States of America; Fred Hutchinson Cancer Research Center, United States of America

## Abstract

[*PSI^+^*], the prion form of the yeast Sup35 protein, results from the structural conversion of Sup35 from a soluble form into an infectious amyloid form. The infectivity of prions is thought to result from chaperone-dependent fiber cleavage that breaks large prion fibers into smaller, inheritable propagons. Like the mammalian prion protein PrP, Sup35 contains an oligopeptide repeat domain. Deletion analysis indicates that the oligopeptide repeat domain is critical for [*PSI^+^*] propagation, while a distinct region of the prion domain is responsible for prion nucleation. The PrP oligopeptide repeat domain can substitute for the Sup35 oligopeptide repeat domain in supporting [*PSI^+^*] propagation, suggesting a common role for repeats in supporting prion maintenance. However, randomizing the order of the amino acids in the Sup35 prion domain does not block prion formation or propagation, suggesting that amino acid composition is the primary determinant of Sup35's prion propensity. Thus, it is unclear what role the oligopeptide repeats play in [*PSI^+^*] propagation: the repeats could simply act as a non-specific spacer separating the prion nucleation domain from the rest of the protein; the repeats could contain specific compositional elements that promote prion propagation; or the repeats, while not essential for prion propagation, might explain some unique features of [*PSI^+^*]. Here, we test these three hypotheses and show that the ability of the Sup35 and PrP repeats to support [*PSI^+^*] propagation stems from their amino acid composition, not their primary sequences. Furthermore, we demonstrate that compositional requirements for the repeat domain are distinct from those of the nucleation domain, indicating that prion nucleation and propagation are driven by distinct compositional features.

## Introduction

**Figure 1:**
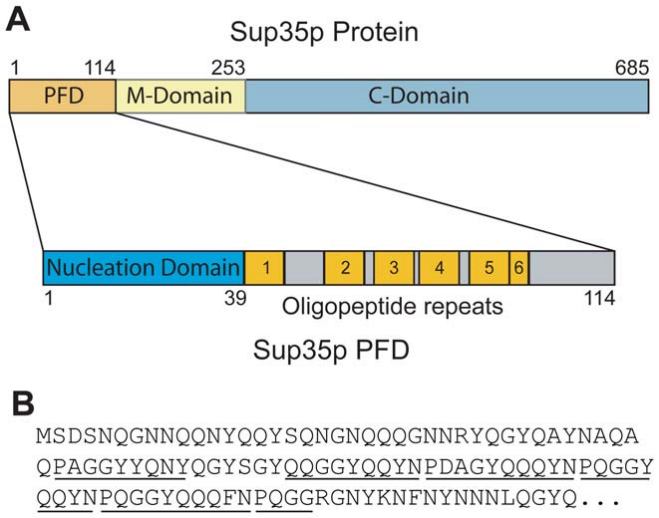
Sup35 layout. (A) Schematic of Sup35. The PFD, the highly charged middle domain (M-domain) and the C-terminal translation termination domain (C-domain) are shown. The PFD is enlarged below, showing the Q/N rich nucleation domain and the oligopeptide repeat domain (ORD). (B) The Sup35 PFD sequence.

The [*PSI^+^*] prion of *Saccharomyces cerevisiae* results from the structural conversion of the Sup35 protein into an infectious amyloid conformation [1], [2], [3]. Because of the rapid growth rate and ease of genetic manipulation of yeast, [*PSI^+^*] has provided a useful model system for examining the mechanism of prion formation and propagation. Sup35 is an essential component of the translation termination machinery in yeast [4], [5]. Sup35 is composed of three functionally and structurally distinct domains (Figure 1): the C-terminal domain (a.a. 254–685) is essential for the translation termination activity, the prion-forming domain (PFD; a.a. 1–114) drives the structural conversion to amyloid, and the highly charged middle domain has no known function other than its ability to stabilize [*PSI* ^+^] fibers [6], [7], [8], [9].

The Sup35 PFD is composed of two separate sub-domains thought to have distinct functions (Figure 1A). The glutamine/asparagine-rich (Q/N-rich) tract (amino acids 1–39) is primarily responsible for prion nucleation and fiber growth, while the remained of the PFD (amino acids 40–114) is thought to be primarily involved in prion propagation [8], [10], [11], [12], [13]; this region dominated by an oligopeptide repeat domain (ORD) spanning amino acids 40–96. The functional delineation between these two sub-domains is not absolute; for example, tyrosine residues in the ORD have recently been implicated in prion nucleation [14]. Nevertheless, a large body of evidence supports an essential role for the ORD in prion propagation.

The ORD consists of 5½ degenerate repeats of the consensus sequence (^P^/_Q_)QGGYQ(^Q^/_S_)YN ([10], [11]; Figure 1B). Truncation of all or part of the ORD [7], [10], [11], [12], or replacement of the repeats with a random sequence [15], destabilizes or eliminates [*PSI^+^*]. In yeast, the chaperone protein Hsp104 is essential for [*PSI^+^*] propagation [16]. Prion aggregates that are too large are not efficiently segregated into daughter cells, resulting in prion loss [17]. Hsp104 cleaves prion fibers, generating new smaller prion “seeds” that can be distributed to daughter cells upon cell division [18], [19], [20]. Because deletion of one or more repeats increases the average [*PSI^+^*] aggregate size, it has been suggested that the repeats facilitate Hsp104-dependent fragmentation, either by acting as a direct binding site for Hsp104 or by changing the conformation of the amyloid core to allow for Hsp104 access [13]. Replacement of the Sup35 ORD in *S. cerevisiae* with the oligopeptide repeat motifs from the Sup35 of *Y. lipolytica*, which has previously been shown to be a potent [*PSI^+^*] forming protein [21], significantly affects prion propagation and allows for prion maintenance in the absence of Hsp104 [15]. Chimeric proteins in which the PFD of Sup35 has been replaced with a poly Q tract (Q62) can form amyloids, but these amyloids are not stably propagated; however, addition of the ORD from Sup35 allows for stable propagation [10]. Therefore, it has been proposed that efficient chaperone-dependent aggregate cleavage may represent the difference between infectious and non-infectious amyloids, and that repeat sequences may play a critical role in allowing for chaperone-dependent cleavage [10].

Although numerous studies have demonstrated that the region encompassing the ORD is important for maintenance of wild-type [*PSI^+^*], none of these studies has examined whether repeats *per se* are required for this function, or whether some other feature of the ORD allows for prion maintenance. Ure2, a highly studied yeast protein responsible for the [URE3] prion, does not contain repeats, demonstrating that repeats are not a necessary feature for prion maintenance. Additionally, four of five scrambled mutants of Sup35 wherein the primary sequence of the PFD was randomized while maintaining amino acid composition were able to stably maintain [*PSI^+^*], suggesting that composition, not primary sequence is the major determinant of the [*PSI^+^*] phenotype [22].

However, these scrambling results have not diminished the attention focused on the ORD. One reason for this continued attention is that similar repeats are found in the mammalian prion protein PrP. The PrP ORD consists of five repeats with a consensus sequence of PHGGGWGQ. Therefore, although PrP and Sup35 are completely unrelated, they both contain repeats that are rich, to varying degrees, in proline, glycine and glutamine (Table 1). The prevalence of proline and glycine are particularly notable, as these residues have low β-sheet and prion propensity [23], [24]. Additionally, the repeats are similar in length (eight versus nine amino acids for PrP and Sup35, respectively) and in number (five versus five and a half copies, respectively).

**Table 1:** Amino acid compositions of Sup35 and PrP fragments.

Fragment^a^	Ala	Arg	Asn	Asp	Cys	Gln	Glu	Gly	His	Ile	Leu	Lys	Met	Phe	Pro	Ser	Thr	Trp	Tyr	Val	Polar^b^
Sup35(1–114)	4.4	1.8	17.5	1.8	0.0	28.1	0.0	16.7	0.0	0.0	0.9	0.9	0.9	1.8	4.4	3.5	0.0	0.0	17.5	0.0	49.1
Sup35(1–39)	7.5	2.5	22.5	2.5	0.0	32.5	0.0	10.0	0.0	0.0	0.0	0.0	2.5	0.0	0.0	7.5	0.0	0.0	12.5	0.0	62.5
Sup35(40–114)	2.7	1.3	14.7	1.3	0.0	26.7	0.0	20.0	0.0	0.0	1.3	1.3	0.0	2.7	6.7	1.3	0.0	0.0	20.0	0.0	42.7
Sup35(65–104)	2.5	2.5	12.5	2.5	0.0	27.5	0.0	20.0	0.0	0.0	0.0	2.5	0.0	5.0	10.0	0.0	0.0	0.0	15.0	0.0	40.0
Human PrP ORD	0.0	0.0	0.0	0.0	0.0	14.6	0.0	51.2	9.8	0.0	0.0	0.0	0.0	0.0	12.2	0.0	0.0	12.2	0.0	0.0	24.4

a Amino acids 1–114 of Sup35 encompass the entire PFD. Amino acids 1–39 are the nucleation domain, while amino acids 40–114 include the entire ORD. Amino acids 65–104 include the last 3½ repeats of the ORD.

b Polar amino acids are Asn, Gln, His, Ser, and Thr.

Expansion of the PrP oligopeptide repeat domain is associated with dominant inherited prion diseases [25], [26], while PrP devoid of the ORD has increased incubation periods and reduced prion titers in terminally ill mice [27]. Due to the similarities of the Sup35 and PrP oligopeptide repeats, Sup35 has been used as a model for examining the role of the PrP repeats in prion formation and propagation [12], [28], [29], [30]. Oligopeptide repeats from PrP can functionally replace the Sup35 ORD in supporting [*PSI^+^*] maintenance [12], and increasing the number of PrP repeats inserted in place of the Sup35 ORD shortens the lag time for in vitro fiber formation assays [30].

Given the similarity between the Sup35 and PrP ORDs, it seems reasonable that the primary sequence of the repeats could be similarly important for some aspects of [*PSI^+^*] formation or propagation. Repeat elements are also found in other yeast prion domains, including those of Rnq1, Mca1 and New1 [10], [31], [32]. Furthermore, a primary sequence element was recently identified within another prion protein, Rnq1, that is required for interaction with the chaperone Sis1p [33], [34]; although this element is not within the repeat domain of Rnq1, it nonetheless bolsters the idea that primary sequence elements can affect chaperone interactions.

There are three general hypotheses that could reconcile the sensitivity of the ORD to deletion with the insensitivity of Sup35 to scrambling. First, the repeats could simply act as a relatively non-specific spacer, separating the nucleation domain from the highly charged M domain. Second, the ORD could be important for its composition, not its primary sequence. Third, Shkundina et al. proposed that the repeats are not necessary in the context of artificial prions such as those formed by scrambled Sup35, but that the repeats may serve a specific function within the context of naturally occurring [*PSI^+^*] [13]. A related theory is that the repeats could explain some unique features of [*PSI^+^*]. [*PSI^+^*] is more sensitive to Hsp104 levels than [*URE3*]; although both [*URE3*] and [*PSI^+^*] are efficiently eliminated by Hsp104 deletion, only [*PSI^+^*] is eliminated by Hsp104 over-expression [35]. Additionally, many spontaneous [*URE3*] isolates are unstable [36], which could be due to the lack of repeats [10].

To distinguish among these hypotheses, we have made a broad range of mutant Sup35 proteins in which the ORD was altered. We found that the primary sequence of the oligopeptide repeats is nonessential for Hsp104-dependent [*PSI^+^*] maintenance, disproving the widely held hypothesis that the oligopeptide repeats provide a specific recognition sequence for chaperone-mediated fiber cleavage. However, the unique composition of the ORD relative to the nucleation domain proved critical for its function.

## Results

### Replacing the ORD with segments from scrambled Sup35 mutant proteins

Because four of five scrambled versions of Sup35 were able to efficiently propagate prions, we hypothesized that each of these proteins must have propagation domains analogous to the ORD. By identifying these regions, we hoped to identify the common features that allow for Hsp104-dependent prion maintenance. Therefore, we replaced the 75 amino acid maintenance domain (a.a. 40–114) of wild-type Sup35, which includes the entire ORD, with either the first 75 amino acids after the start codon (amino acids 2–76) or the last 75 amino acids (amino acids 40–114) of the PFDs from the scrambled Sup35 mutants (Sup35-21, -24, -25, -26 and -27) [22]. The fusion constructs were named FP21N (fusion protein Sup35-21 N-terminus, indicating that the ORD of wild-type Sup35 was replaced with the N-terminal 75 amino acids from the scrambled prion protein Sup35-21), FP21C, FP24N, FP24C, FP26N, FP26C, FP27N and FP27C (complete sequences can be found in the supplemental information – see Text S1). Each was expressed from a low-copy (*CEN*) plasmid under the *S. cerevisiae SUP35* promoter.

Each fusion construct was tested in a *S. cerevisiae* strain lacking chromosomal *SUP35* and expressing wild-type *SUP35* from a *URA3* plasmid. Using plasmid shuffling, each construct was introduced into a [*PSI^+^*] strain in the place of the endogenous *SUP35*. Each strain was then assayed for [*PSI^+^*] propagation by monitoring nonsense suppression of the *ade2-1* allele [37]. In the absence of [*PSI^+^*], *ade2-1* mutants are unable to grow without adenine and form red colonies in the presence of limiting adenine due to accumulation of a pigment derived from the substrate of Ade2p. [*PSI^+^*] causes stop-codon read-through, allowing for growth without adenine and white or pink colony formation in the presence of limiting adenine.

**Figure 2:**
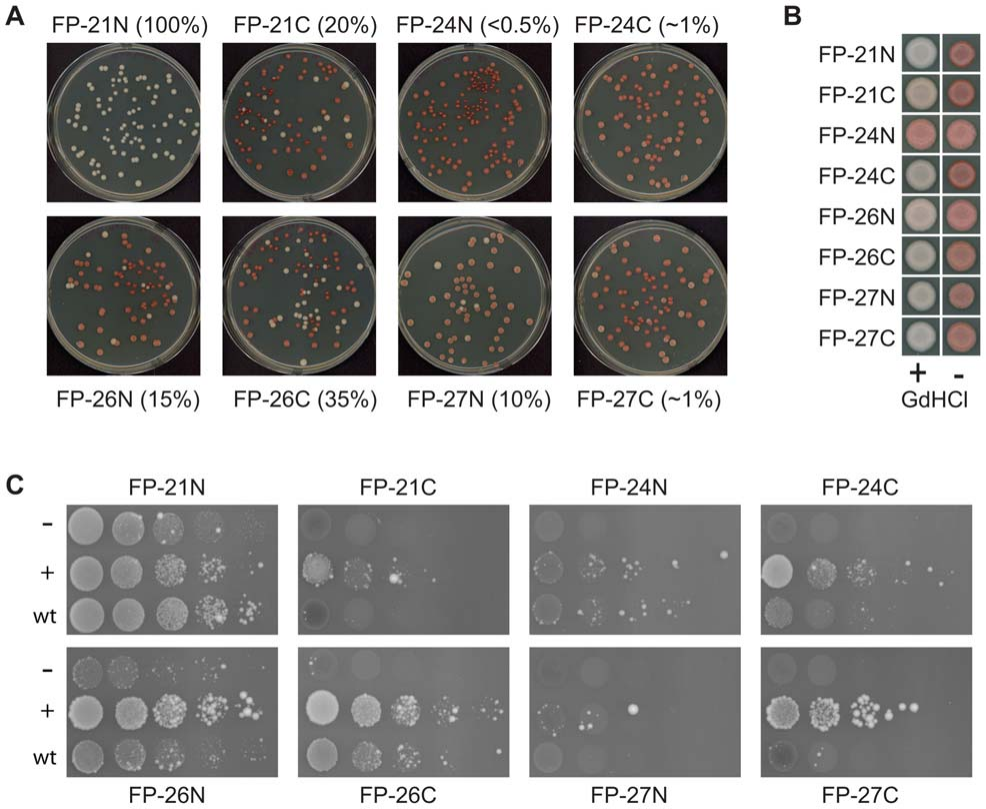
Fusion proteins maintain wild type [*PSI^+^*]. (A) Molecular compatibility between wild-type [*PSI^+^*] aggregates and fusion proteins in which the ORD was replaced with fragments from the scrambled PFDs. Fusion proteins were introduced into a wild-type [*PSI^+^*] strain by plasmid shuffling and then plated for single colonies on YPD to test for [*PSI^+^*] by color phenotype. Approximate percentages of [*PSI^+^*] cells for each strain are indicated in parentheses. (B) [*PSI^+^*] stability and curing. White colonies expressing the fusion proteins were streaked for single colonies on YPAD or YPAD plus 4 mM guandine HCl and then spotted onto YPD to test for [*PSI^+^*]. Because FP-24N did not form any white colonies, guandine HCl treatment of a red colony is shown. (C) Strains expressing the fusion proteins were transformed with either the empty vector pKT24 (−) or with a derivative pKT24 expressing from the *GAL1* promoter either the NM domain of the same variant of *SUP35* (+) or the NM domain of wild-type Sup35 (wt). Strains were grown in galactose/raffinose dropout medium and serial dilutions plated onto medium lacking adenine to select for [*PSI^+^*].

Surprisingly, only one of the eight fusion mutants, FP-24N, appeared completely unable to propagate the Ade^+^ phenotype. When FP-24N was introduced into a wild-type [*PSI^+^*] strain in the place of the endogenous *SUP35*, the vast majority of the colonies were red on limiting adenine, indicating a loss of [*PSI^+^*] (Figure 2A). Although rare pink colonies were observed, when these were restreaked onto limiting adenine, the majority of the resulting colonies were red (data not shown), demonstrating an inability to maintain the [*PSI^+^*] phenotype.

By contrast, FP-21N showed 100% white colonies (Figure 2A). To confirm that maintenance of the Ade^+^ phenotype was a result of [*PSI* ^+^] maintenance, we tested whether the Ade^+^ phenotype could be cured by treatment with low concentrations of guanidine HCl. Guanidine HCl cures [*PSI* ^+^] [38] by inhibiting Hsp104 [39], [40], [41]. In all cases, the Ade^+^ phenotype was efficiently cured by treatment with 4 mM guanidine HCl, demonstrating that FP-21N was indeed maintaining [*PSI^+^*] in an Hsp104-dependent manner (Figure 2B).

The remaining six constructs showed a mixture of red and white colonies. In all cases, the white colonies reverted to the red phenotype upon treatment with guanidine HCl, indicating that the phenotype was the result of a prion (Figure 2B). There are two possible explanations for the observed mixture of red and white colonies. First, the mutants might not efficiently add onto the pre-existing wild-type [*PSI^+^*] aggregates due to their sequence differences with wild-type Sup35. This molecular incompatibility is analogous to the species barrier seen for the mammalian prion proteins, in which prion transmission is inefficient between different species due to primary sequence differences between the proteins. Second, the mutants might have some defect in prion propagation resulting in prion loss; for example, the mutants might not be efficiently recognized by cellular chaperones involved in prion propagation.

To distinguish between these two possibilities, for each of the six mutants that showed a mixture of red and white colonies, white colonies were re-streaked to test for stability of the Ade^+^ phenotype. In each case, no detectable prion loss was observed even upon multiple passages on non-selective medium (data not shown). Sup35 was sequenced from these cells to ensure that prion propagation was not a result of recombination during plasmid shuffling between the plasmids expressing wild-type and mutant Sup35; in all cases colonies were identified which stably maintained [*PSI^+^*] while expressing only the mutant Sup35 (data not shown). This demonstrates that the mutants can efficiently propagate prions and suggests that the red colonies observed in Figure 2A were due to molecular incompatibility.

Since prion formation occurs by a spontaneous molecular conversion event, increasing the number of molecules increases the likelihood of prion formation. To further examine this molecular incompatibility, we tested the ability of each mutant to form Ade^+^ colonies upon transient overexpression of either its matching PFD domain or the wild-type PFD domain (Figure 2C). For all mutants tested, Ade^+^ colony formation was detectable, and increased with overexpression. Overexpression of the wild-type PFD domain and the FP21N PFD domain induced prion formation by full-length FP21N with comparable efficiencies (Figure 2C), consistent with the lack of a molecular incompatibility barrier in the plasmid shuffling assays (Figure 2A). By contrast, all other mutants except FP24N were induced significantly less efficiently by the wild-type NM domain than by the matching domain. Additionally, for all mutants except FP24N, the Ade^+^ phenotype in majority of the Ade^+^ colonies was curable by guanidine HCl, but was stably maintained in the absence of guanidine HCl, confirming that the Ade^+^ phenotype was the result of [*PSI* ^+^] formation (data not shown).

FP24N was induced with roughly comparable efficiencies by the wild-type and FP24N NM domains. In both cases, the majority (80–90%) of the Ade^+^ colonies were unstable, showing rapid conversion to an Ade^−^ phenotype. The rare stable Ade^+^ colonies were consistently curable by guanidine HCl. Thus, even FP24N, which was completely unable to propagate wild-type [*PSI^+^*], was able to form rare stable prions de novo.

Therefore, despite their divergent compositions (Table 2) seven of the eight mutants tested were able to efficiently propagate wild-type [*PSI^+^*], although all but one first had to overcome some degree of molecular incompatibility. Only FP24N showed a significant prion propagation defect, and even this mutant was able to rarely form stable, self-propagating prions. These results demonstrate the broad sequence requirements for the ORD region.

**Table 2:** Composition of residues 40–114 in each of the FP constructs.

	Ala	Arg	Asn	Asp	Cys	Gln	Glu	Gly	His	Ile	Leu	Lys	Met	Phe	Pro	Ser	Thr	Trp	Tyr	Val	Polar^a^
FP-21N	4.0	1.3	18.7	2.7	0.0	26.7	0.0	18.7	0.0	0.0	0.0	1.3	0.0	2.7	2.7	4.0	0.0	0.0	17.3	0.0	49.3
FP-21C	5.3	1.3	18.7	0.0	0.0	25.3	0.0	18.7	0.0	0.0	1.3	1.3	0.0	1.3	6.7	1.3	0.0	0.0	18.7	0.0	45.3
FP-24N	4.0	2.7	20.0	1.3	0.0	28.0	0.0	13.3	0.0	0.0	0.0	1.3	0.0	2.7	4.0	4.0	0.0	0.0	18.7	0.0	52.0
FP-24C	5.3	0.0	13.3	2.7	0.0	32.0	0.0	18.7	0.0	0.0	1.3	1.3	0.0	2.7	2.7	2.7	0.0	0.0	17.3	0.0	48.0
FP-26N	2.7	2.7	16.0	0.0	0.0	32.0	0.0	13.3	0.0	0.0	1.3	0.0	0.0	1.3	5.3	2.7	0.0	0.0	22.7	0.0	50.7
FP-26C	5.3	1.3	18.7	2.7	0.0	25.3	0.0	18.7	0.0	0.0	0.0	1.3	0.0	2.7	4.0	2.7	0.0	0.0	17.3	0.0	46.7
FP-27N	2.7	2.7	16.0	1.3	0.0	30.7	0.0	16.0	0.0	0.0	0.0	1.3	0.0	1.3	4.0	5.3	0.0	0.0	18.7	0.0	52.0
FP-27C	6.7	2.7	18.7	1.3	0.0	28.0	0.0	17.3	0.0	0.0	1.3	0.0	0.0	1.3	6.7	1.3	0.0	0.0	14.7	0.0	48.0

Shown are the compositions for the 75 amino acid sequences used to replace the ORD (residues 40–114) in each of the FP constructs.

a Polar amino acids are Asn, Gln, His, Ser, and Thr.

### Scrambling the ORD of Sup35

Although deletion of even one repeat inhibits efficient prion propagation, only the first two repeats are required for efficient addition to existing prion aggregates in vivo [10], [12], [13]. To separately examine the primary sequence requirements of the ORD region for [*PSI^+^*] propagation and molecular compatibility, we randomized the order of the amino acids in either all of the repeats or just the last 3½ repeats while keeping amino acid composition constant. In both cases, three scrambled constructs were generated (see supplemental information for sequences).

**Figure 3:**
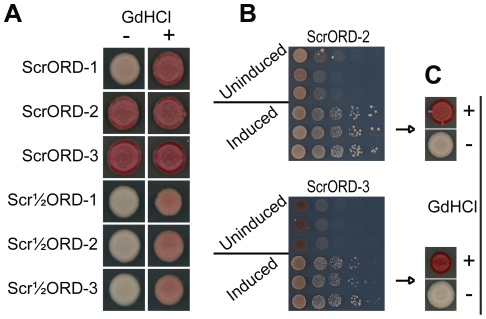
Scrambling the ORD does not prevent prion maintenance. (A) Maintenance of [*PSI^+^*] by *SUP35* mutants in which the primary sequence the entire ORD or the last 3½ repeats of the ORD was scrambled. Plasmids expressing mutant versions of *SUP35* were introduce into a wild-type [*PSI^+^*] strain by plasmid shuffling. After testing for prion maintenance on YPD, individual white colonies were streaked onto YPD or YPD plus 4 mM guanidine HCl and then spotted onto YPD to test for [*PSI^+^*]. Because no white colonies were observed for ScrORD-2 and -3 after counterselection, pink colonies were tested. (B) Scrambled ORD Sup35 mutants tested for de novo prion formation with (induced) and without (uninduced) overexpression of the matching NM domain. (C) Ade^+^ colonies induced by overexpression of ScrORD-2 and ScrORD-3 were grown on YPAD with and without 4 mM GdHCl and tested for [*PSI^+^*].

All three of the mutants in which the last 3½ repeats were scrambled (Scr½ORD1, 2 and 3) showed no molecular incompatibility with wild-type [*PSI^+^*] aggregates and efficiently maintained [*PSI^+^*] without any detectable prion loss (Figure 3A and data not shown). This demonstrates that the primary sequence of the last 3½ repeats plays little or no role in molecular compatibility or efficiency of prion propagation. By contrast, scrambling all 5½ repeats resulted in significant molecular incompatibility between the scrambled mutants and wild-type [*PSI^+^*] aggregates. Of the three constructs in which all 5½ repeats were scrambled (ScrORD1, 2 and 3), only one mutant, ScrORD-1, overcame this molecular incompatibility at a detectable frequency (Figure 3A). For ScrORD-1, overcoming this barrier was a rare event; however, once overcome, maintenance of the mutant prion was indistinguishable from wild type [*PSI^+^*] propagation (data not shown). Although the ScrORD-2 and ScrORD-3 mutant proteins were completely incompatible with wild-type [*PSI^+^*] aggregates, they could be induced to form stable, curable prions (Figure 3B and 3C), demonstrating that their failure to propagate wild type [*PSI* ^+^] was a result of molecular incompatibility, not an intrinsic inability to propagate prions.

Thus, the primary sequence of the first two repeats is an important determinant of molecular compatibility, consistent with results from in vitro experiments indicating that the first two repeats are involved in critical contacts within the core of Sup35 amyloid fibers [42]. However, once this incompatibility barrier is overcome, the repeats per se are not required for [*PSI^+^*] maintenance.

### Hsp104 over-expression cures scrambled prions

Although repeats are clearly not an absolute requirement for prion formation and propagation, the presence of repeats in Sup35 could exert a more subtle effect; for example, because of the repeat region's role in Hsp104-dependent prion propagation, the repeats could explain why, unlike [URE3], [*PSI^+^*] is cured by Hsp104 overexpression. Consistent with a subtle role of repeat elements in prion formation or propagation, scrambled versions of Sup35 show broadly varying rates of prion formation, strengths of the prion phenotype and efficiencies of prion maintenance [22].

**Figure 4:**
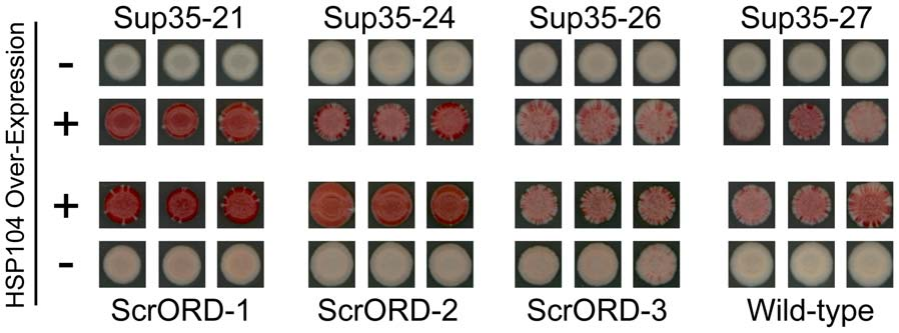
Hsp104 overexpression cures Sup35 ORD mutants. [*PSI^+^*] strains expressing scrambled versions of *SUP35*, scrambled ORD mutants or wild-type *SUP35* were transformed either with a plasmid expressing *HSP104* from the *ADH1* promoter (+) or an empty vector. After 3 days growth, transformed strains were spotted onto YPD to test for [*PSI^+^*].

To determine whether the primary sequence of the ORD is responsible for the unique sensitivity of [*PSI^+^*] to Hsp104 overexpression, we tested the effects of Hsp104 overexpression on the scrambled mutants. [*PSI^+^*] cells expressing Sup35 mutants in which either the entire PFD [22] or the ORD was scrambled were transformed with a plasmid that constitutively over-expresses *HSP104* from an *ADH1* promoter. After three days of growth, the transformed colonies were tested for prion loss (Figure 4). Although prion curing is accomplished with varying efficiency for each mutant, they are all sensitive to Hsp104 overexpression despite their lack of oligopeptide repeats. Clearly, the primary sequence of the ORD is not responsible for the sensitivity of [*PSI^+^*] aggregates to Hsp104 overexpression.

### Replacing the ORD with a scrambled Sup35 nucleation domain

Although the last 3½ repeats of the Sup35 ORD are required for efficient prion maintenance, they are not required for prion nucleation or for molecular compatibility with wild-type Sup35 prion aggregates ([10]; Figure 3). Therefore, this region provides the perfect system for examining the specific sequence/composition requirements for prion maintenance.

**Figure 5:**
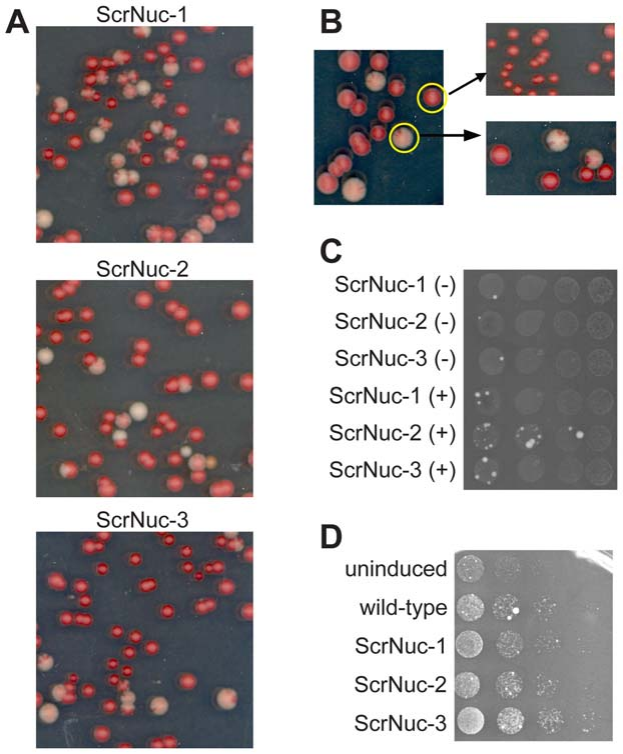
ScrNuc mutants do not support [*PSI^+^*] propagation. (A) Molecular incompatibility between wild-type [*PSI^+^*] aggregates and *SUP35* mutants in which the last 3½ repeats of the ORD was replaced with a scrambled Sup35 nucleation domain. Fusion proteins were introduced into a wild-type [*PSI^+^*] strain by plasmid shuffling and then plated for single colonies on YPD to test for [*PSI^+^*] by color phenotype. (B) White and red colonies from a strain expressing ScrNuc3 were restreaked onto YPD to test for stability of the red or white phenotype. (C) ScrNuc mutants fail to form Ade^+^ colonies de novo. ScrNuc mutants were tested for de novo prion formation with (−) and without (+) overexpression of the matching NM domain. To allow for detection of slow-growing Ade^+^ colonies, plates were grown for ten days instead of the standard six days. (D) Overexpression of the ScrNuc PFDs induces wild-type [*PSI^+^*] formation. Yeast expressing full-length wild-type Sup35 were transformed with an empty vector (uninduced), or with a plasmid overexpressing either wild-type Sup35 (wild-type) or one of the ScrNuc (ScrNuc-1, -2 and -3) PFDs. Cells were grown in galactose/raffinose dropout medium and serial dilutions plated onto medium lacking adenine to select for [*PSI^+^*].

To determine whether specific compositional elements within the ORD are critical for prion maintenance, we asked whether the nucleation domain could substitute for the ORD in promoting maintenance. We replaced the last 3½ repeats of the ORD with a scrambled version of the Sup35 PFD nucleation domain (see supplemental information for sequences). Interestingly, none of the resulting mutant proteins (ScrNuc1, 2 and 3) were capable of efficiently maintaining [*PSI^+^*]. When plasmid shuffling was used to replace wild-type Sup35 in a [*PSI^+^*] strain, the majority of colonies showed significant sectoring, indicating high rates of [*PSI^+^*] loss (Figure 5A). In each case a small fraction of the colonies were fully red, indicating prion loss, and a small fraction were white. When the red colonies were re-streaked for single colonies, all of the resulting colonies were red; by contrast, when the white colonies were re-streaked, again mostly sectored colonies were observed (Figure 5B). Even after multiple rounds of selecting rare white colonies and re-streaking, no stable white colonies were ever identified (data not shown). Thus, the ScrNuc constructs are clearly unable to efficiently propagate wild-type [*PSI^+^*].

This failure to propagate wild-type [*PSI^+^*] could reflect a general inability of the mutants to propagate prions, or it could simply reflect an inability to propagate the specific prion variant initially present in the yeast strain. We repeated the plasmid shuffling assay in strains containing a number of independently derived weak and strong prion variants; in all cases, the ScrNuc constructs were unable to propagate the prions (data not shown). Additionally, when cells expressing ScrNuc1, 2 or 3 were plated onto SC-ade medium to select for spontaneous prion formation, very few Ade^+^ colonies were formed with or without prion domain overexpression, even after ten days of incubation (Figure 5C); none of the Ade^+^ colonies tested for any of the three mutants were both stably Ade^+^ and guanidine HCl-curable (data not shown). Thus, the ScrNuc mutants are not able to propagate wild-type [*PSI^+^*] aggregates, and do not form their own stably propagating aggregates de novo at a detectable frequency. This demonstrates that the composition of the ORD is critical for its ability to support [*PSI* ^+^] propagation. However, the PFDs from the ScrNuc mutants were able to induce wild-type [*PSI* ^+^] when overexpressed in cells expressing full-length wild-type Sup35 (Figure 5D). This suggests that these mutants maintain the ability to form aggregates, but are simply unable to stably propagate these aggregates.

### Replacing the ORD with scrambled PrP repeats

Mutagenesis analysis suggests that amyloid formation by Q/N-rich proteins is driven by fundamentally different sequence features than amyloid formation by non-Q/N-rich proteins. Amyloid formation by non-Q/N-rich proteins is thought to be driven predominantly by short, highly amyloidogenic segments [43] while amyloid formation by Q/N-rich proteins seems to be driven by the sum of many weaker interactions across a large, intrinsically disordered domain [24], [44]. Amyloid formation by non-Q/N-rich proteins also appears to be more primary sequence dependent; unlike scrambling of the Q/N-rich proteins Sup35 and Ure2, scrambling of various non-Q/N-rich amyloid proteins seems to block amyloid formation [45], [46]. PrP is not Q/N rich, which raises the question: Is the prion-promoting activity of the PrP repeats primary sequence independent?

**Figure 6:**
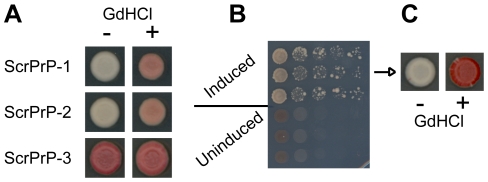
ScrPrP mutants maintain wild type [*PSI^+^*]. (A) Maintenance of [*PSI^+^*] by fusion proteins in which the last 3½ repeats of the ORD was replaced with scrambled PrP ORDs. (B) De novo prion formation for ScrPrP-3, with (induced) and without (uninduced) overexpression of the matching PFD. (C) Curability of the Ade^+^ phenotype in the scrambled PrP mutants.

To address this question, we generated three constructs (ScrPrP-1, 2 and 3) in which the last 3½ repeats of Sup35 were replaced with a scrambled version of the human PrP ORD (see supplemental information for sequences). Two of the three constructs were capable of maintaining [*PSI^+^*] with no molecular incompatibility (Figure 6A and data not shown). ScrPrP-3 was incompatible with the wild-type [*PSI^+^*] prion, but was able to form prions de novo (Figure 6B), demonstrating that sequences in the final 3½ repeats can, under rare circumstances, create a species barrier. Once formed, these prions were maintained efficiently in an Hsp104-dependent manner, with no detectable prion loss (Figure 6C and data not shown). Therefore, the ability of the PrP repeats to substitute for the Sup35 repeats in promoting [*PSI^+^*] propagation is not dependent on the primary sequence of the repeats.

## Discussion

A large body of literature highlights the importance of the Sup35 ORD for [*PSI^+^*] maintenance [10], [11], [12], [13], [15], [29], [30]. The presence of repeat elements in many other prion proteins, including Prp, Mca1, New1 and Rnq1 seems to support the importance of repeats. However, other yeast prion-forming proteins such as Ure2 are capable of forming and propagating prions without oligopeptide repeats, and scrambling the Sup35 PFD does not block [*PSI^+^*] formation or propagation [22]. We sought to address this disconnect.

Our experiments clearly show that the ORD is important not for its primary sequence. While this result might be expected due to Sup35's insensitivity to scrambling, it remained possible that the repeats either were required to propagate wild-type [*PSI^+^*] [13] or explained some unique characteristic of [*PSI^+^*] such as sensitivity to Hsp104 overexpression. Our results show neither of these to be true. While deletion of even a single repeat significantly reduces [*PSI^+^*] mitotic stability [13], scrambling all of the repeats did not consistently reduce the efficiency of prion formation, propagation or sensitivity to Hsp104 overexpression. Therefore, although there are subtle differences in prion formation and Hsp104 sensitivity among our ScrORD mutants (and there may be other subtle differences that we have not detected), clearly the activity of the ORD is largely primary sequence independent.

However, the inability of the ScrNuc constructs to propagate prions demonstrates that the distinct composition of the ORD (relative to the nucleation domain) is critical to its functions. This result was surprising for two reasons. First, the fact that eight fragments tested from the scrambled versions of Sup35 were able to support prion propagation seemed to suggest that the sequence requirements for the ORD region were highly flexible. Second, additional repeats can substitute for the nucleation domain in supporting prion formation [29], which would seem to suggest that the two domains are interchangeable; instead, the fact that the reverse is not true argues that the compositional requirements for nucleation are more flexible than for propagation.

Although the composition of the ORD appears to be critical for its ability to support [*PSI^+^*] propagation, it is unclear what aspects of composition are important. Other studies have looked at the overall compositional requirements for yeast prions [24], [44]. However, these studies did not separately look at the compositional requirements for formation versus propagation. Therefore, defining the distinct compositional requirements for these two activities will be important. Alexandrov et al. previously proposed that tyrosine residues may specifically promote fiber fragmentation [47]; however, the ScrNuc constructs have nearly identical tyrosine content to wild-type Sup35, with twenty tyrosines in wild-type Sup35 PFD versus nineteen in ScrNuc PFDs. By contrast, the nucleation domain does have considerably more polar residues and fewer glycine and proline residues than either the PrP or Sup35 ORDs (Table 1), raising the possibility that some combination of these compositional features could be important for prion propagation. Intriguingly, FP24N, the only fusion protein from Figure 2 that shows a significant propagation defect, is tied for both the fewest glycines and most polar residues of the eight fusion proteins (Table 2).

In scrambling the primary sequence of the ORD, we found that repeats 1 and 2 are important determinants for molecular compatibility between the wild-type prion and the mutant proteins, while repeats 3 through 5½ have only a weak affect on molecular compatibility. This observation is consistent with in vitro data that suggest repeats 1 and 2 are within the core of the amyloid fiber [42], and in vivo data that implicates these repeats being important to fiber formation [11]. However, the primary sequence of these repeats is not critical to the integrity of the fiber; once mutants with repeats 1 and 2 disrupted overcame the molecular incompatibility with wild-type [*PSI^+^*], the prion was propagated in a manner indistinguishable from wild-type. Additionally, even for the first two repeats, the primary sequence is not absolutely required for molecular compatibility with wild-type. Two constructs in which we disrupted the first two repeats (FP-21N and ScrORD-1) showed full molecular compatibility with wild-type [*PSI^+^*]. There is not a single residue in the first two repeats of Sup35 that is conserved in both FP21N and ScrORD-1, nor does any portion contain obvious sequence similarity, clearly demonstrating the lack of stringent primary sequence requirements. Interestingly, there is also no obvious compositional feature within this region that separates FP21N and ScrORD-1 from the other FP and ScrORD constructs. Thus, it is possible that this region must just be structurally compatible with wild-type [*PSI^+^*] to mediate molecular compatibility, rather than requiring specific sequence features.

Although our results appear to conflict with the wide body of literature supporting the importance of the ORD, this difference may be due to differences in the methods employed; past experiments concluding the ORD is important to [*PSI^+^*] propagation were largely based on examination of Sup35 mutants containing either truncated or expanded ORDs. The problem with such studies is that in addition to changing the number of repeats, truncating or expanding the ORD also changes the total length of the PFD, as well as the spacing between the nucleation domain and the Sup35 C-terminus, making it easy to misinterpret the basis for the observed effects.

Examining the compositional requirements for the ORD may provide insight into the basis for Hsp104 recognition of prion aggregates. Although it has long been known that [*PSI^+^*] is uniquely sensitive to Hsp104 overexpression, the basis for this effect is unknown [48]. Hsp104 has been proposed as a tool to combat amyloid-based neurodegenerative disorders [49]; therefore, determining why some amyloids are completely eliminated by high levels of Hsp104 is important. The simplest explanation is that differences in sensitivity to Hsp104 overexpression are due to differences in the physical properties (such as fiber stability or growth rate) of the aggregates formed by different proteins. However, the scrambled versions of Sup35 have significantly different efficiencies of prion formation and propagation, yet all are cured by Hsp104 overexpression. This suggests that the sequence features that allow for [*PSI^+^*] curing by overexpression are distinct from those that determine rates of prion formation and strength of the prion phenotype.

While our results clearly show that the composition of the ORD is critical for [*PSI^+^*] propagation, questions remain regarding the molecular basis for this activity. Based on the variety of evidence suggesting that the ORD promotes Hsp104-dependent aggregate cleavage [10], [12], [13], it was proposed that the repeats may facilitate [*PSI^+^*] propagation by acting as an Hsp104 binding site [10], [13]. Alternatively, the ORD could affect the conformation of the fibril, improving access of Hsp104 to the fibril core [10], [13]. Our results do not fully distinguish between these hypotheses. While our results demonstrate that the repeats do not act as a primary-sequence-specific Hsp104 binding site, it remains possible that compositional elements within the repeats allow for Hsp104 binding; given that Hsp104 can recognize prion proteins with diverse sequences, it would not be surprising for its binding to be primary sequence independent. Alternatively, just as the Sis1 binding site within Rnq1 is outside of the PFD [34], Hsp104 may bind outside the Sup35 PFD, with the composition of the ORD influencing the fiber structure to facilitate access of Hsp104 to the fiber core.

If primary sequence has little effect on prion formation and propagation, why are repeats found in several prion-forming proteins? Analysis of the sequence requirements for prion formation suggests that yeast prion domains are characterized by long disordered regions of modest prion propensity, rather than short regions of high prion propensity [24]. Repeat expansion provides a simple mechanism for generating such regions. The simplest way to generate a long disordered region of modest prion propensity would be to duplicate a short disordered region of modest prion propensity. Thus, we propose that repeats are found in many yeast prion domains not because repeat sequences affect the biochemistry of prion formation or propagation, but instead because they provide a genetic mechanism for domain expansion, and therefore prion domain generation.

Prior to this work, we viewed the ability of the PrP ORD to substitute for the Sup35 ORD with some skepticism; it seemed possible that the Sup35 ORD might simply act as a relatively non-specific spacer, promoting prion propagation by separating the nucleation domain from the charged M domain. If, for example, any relatively uncharged segment could serve this function, the ability of the PrP repeats to substitute for the Sup35 repeats would not be particularly meaningful, and might not reveal anything about the function of the repeats within PrP. However, our observation that compositional changes in the ORD region can block prion propagation makes the ability of the PrP repeats to substitute for the Sup35 ORD more meaningful, and furthers the connection between Sup35 and PrP repeats. Additionally, the primary sequence insensitivity of the PrP ORD is noteworthy, as this is, to our knowledge, the first example of a non-Q/N-rich amyloid-promoting domain showing primary-sequence independence. It remains possible that the primary sequence of the ORD has some prion-promoting function within the context of PrP. However, these results raise the possibility that the disease-promoting effects of PrP ORD expansions [50], [51] may not be due to the actual repeats *per se*, but due to the fact that expansion of a disordered, aggregation-prone region is likely to increase amyloid-forming propensity.

## Materials and Methods

### Strains and media

Standard yeast media and methods were used as previously described [52], except the YPD contained 0.5% yeast extract instead of the standard 1%. In all experiments, yeast were grown at 30°C. All experiments were performed in *Saccharomyces cerevisiae* strain 780-1D/pJ533 [53]. This strain's genotype is *α kar1-1 SWQ5 ade2-1 his3 leu2 trp1 ura3 sup35*::KanMx [PSI^+^] [PIN^+^]; pJ533 expresses *SUP35* from a *URA3* plasmid as the sole copy of *SUP35* in the cell.

### Replacing the Sup35 ORD

FP21C, FP24C, FP26C, and FP27C were constructed in two steps. Fragments from the scrambled Sup35s were amplified by PCR using primer EDR262 paired with a construct-specific primer (see Supplemental Table S1 for all oligonucleotide sequences). The N-terminal portion of the PFD was amplified in a separate reaction from pJ533 using primers EDR257 and EDR313. N-terminal and scrambled fragments were combined and reamplified with EDR259 and EDR261. PCR products were co-transformed with AatII/HindIII-cut pJ526 (from Dan Masison, National Institutes of Health) into yeast strain 780-1D/pJ533. Transformants were selected on SC-leu and then stamped onto 5-fluoroorotic acid (5-FOA) containing medium to select for loss of pJ533. Plasmids expressing mutant *SUP35*s were confirmed by DNA sequencing.

FP21N, FP24N, FP26N, and FP27N were assembled from three pieces. In separate reactions, the N-terminal portion of the prion domain and the M domain were amplified from pJ533 using primer pairs EDR257/313 and EDR322/262, respectively. Fragments from the scrambled Sup35s were amplified by PCR using construct-specific primers; these products were combined with the N-terminal and M-domain PCR products, re-amplified with EDR259 and EDR261, and cloned into pJ526.

The scrambled portion of the ScrORD constructs was built using six overlapping oligonucleotides (EDR896, EDR236, EDR243, and three construct-specific oligonucleotides). In a separate reaction, the N-terminal portion of the PFD was amplified from pJ533 using primers EDR348 and EDR897. The N-terminal and ORD fragments were combined, re-amplified with EDR869 and EDR871, and cloned into pJ526.

For each of the ScrPrP and Scr½ORD constructs except for ScrPrP2, the scrambled portion of the prion domain was built using four overlapping oligonucleotides (EDR896, EDR236 and two construct-specific oligonucleotides). In a separate reaction, the N-terminal portion of the PFD was amplified from pJ533 using primers EDR348 and EDR870. The N-terminal and ORD fragments were combined, re-amplified with EDR348 and EDR243, and cloned into pJ526. ScrPrP2 was built like the ScrORD constructs, using six overlapping oligonucleotides (EDR947, 948, 893, 896, 236, and 243) to assemble the scrambled portion.

The ScrNuc constructs were assembled from separate N- and C-terminal PCR reactions. The N-terminal portion was amplified from pJ533 using EDR257, paired with EDR1025, 1030 or 1034, respectively. The C-terminal portion of ScrNuc2 was amplified from pJ533 using EDR305 paired with EDR1031. The C-terminal portions of ScrNuc-1 and -3 were generated in two steps. First, EDR305 and EDR1027 were used to PCR amplify from pJ533; this product was then re-amplified with EDR262, paired with either EDR1026 or EDR1035. For each ScrNuc construct, the N- and C-terminal fragments were combined, re-amplified with EDR259 and EDR261, and cloned into pJ526.

### Testing for prion maintenance and curing

Plasmids expressing Sup35 mutants were transformed into strain 780-1D/pJ533. Transformed colonies were re-suspended in water in a 96-well microtiter plate, spotted onto minimal media plates containing 5-FOA and grown for 2–3 days at 30°C to select for loss of pJ533. Cells from the 5-FOA plates were streaked onto YPD plates to test for [*PSI^+^*].

To test for curability, white Ade^+^ colonies were grown on YPAD or YPAD plus 4 mM guanidine HCl (GdHCl). Single colonies were spotted onto YPD to test for loss of [*PSI^+^*].

### Induction experiments

To generate induction plasmids, the N and M domains of the mutant *SUP35*s were amplified by PCR with primers EDR1008 and EDR969, installing a *Bam*HI site before the start codon, and a stop codon and *Pst*I site after the M domain. PCR products were digested with *Bam*HI and *Pst*I and inserted into *Bam*HI/*Pst*I-cut pKT24 (from Kim Taylor, NABI, Rockville, MD), which contains the *GAL1* promoter [54]. Ligation products were transformed into *Escherichia coli* and analyzed by DNA sequencing. Induction experiments were performed as previously described [54]. The wild-type induction plasmid, pER161, was previously described [54]. All plates were grown for six days, except the ScrNuc inductions, which were grown for ten.

### Prion curing by Hsp104 over-expression

Strains were transformed with pER10, which expresses *HSP104* from the *ADH1* promoter or a vector control (pER41). Transformed colonies were re-suspended in water in a 96-well microtiter plate, spotted onto YPD to test for loss of [*PSI^+^*].

## Supporting Information

Table S1 Oligonucleotides used in this study.
(DOC)

Text S1 Supplemental information – Protein sequences.
(DOC)
