# Supplementary material for: [PSI+] Maintenance Is Dependent on the Composition, Not Primary Sequence, of the Oligopeptide Repeat Domain
Source: PLoS One. 2011 Jul 8;6(7):e21953. doi: 10.1371/journal.pone.0021953 (PMC3132755; doi:10.1371/journal.pone.0021953)
Supplement: Table S1 — Oligonucleotides used in this study. (DOC) [file pone.0021953.s001.doc]

Table S1: Oligonucleotides used in this study

| **Primer Name** | **Construct** | **Sequence** |
| --- | --- | --- |
| EDR319 | FP21C | gttatcaagcttacaatgctcaagccGGCCAATACTACCCTCAGG |
| EDR447 | FP24C | gttatcaagcttacaatgctcaagccTATCAAAATCAATACTACCAACAGGCTGG |
| EDR448 | FP26C | gttatcaagcttacaatgctcaagccTATCCCCAATACTACTACGGAAATC |
| EDR451 | FP27C | gttatcaagcttacaatgctcaagccAATTACCAACAAGCTGGAGGTTAC |
| EDR315 | FP21N | gttatcaagcttacaatgctcaagccTCGCAATATAATTCTTACAATGGTCAACAACAACAATTCC |
| EDR316 | FP21N | gagactgtggttggaaaccagcGAACCCGTTATTGGCGTAGC |
| EDR445 | FP24N | gttatcaagcttacaatgctcaagccTCGAATAATCAGAATGGCCAACC |
| EDR446 | FP24N | gagactgtggttggaaaccagcTTGGTTCTGGTACTGTGCCTGTTGGAAC |
| EDR449 | FP26N | gttatcaagcttacaatgctcaagccTCGTATCAATATCAAAACAACTACGGTAATC |
| EDR450 | FP26N | gagactgtggttggaaaccagcATTGCCGTAGAAATTCTGGTTCTGAC |
| EDR452 | FP27N | gttatcaagcttacaatgctcaagccTCGTATCAGGGTTACCAAAATGG |
| EDR453 | FP27N | gagactgtggttggaaaccagcGGGACGTTGATATTGTTGCTGTTGTGAC |
| EDR872 | ScrORD1 | GTTATCAAGCTTACAATGCTCAAGCCGCATTCCAACAACAACCATATGGCCAAAATTATCCTTACGGATACTCTTATGCCCAGC |
| EDR873 | ScrORD1 | CGGATACTCTTATGCCCAGCAAGGTCAGTATCAATACGGGTATAATCAAGACTACTACAATAATCCTTACGGCTACGGTCAGGGTGGTGGTCAAC |
| EDR874 | ScrORD1 | GTTGAAGTTTTTGTAATTTCCACGGCCTTGCTGTTGATTCCCCTGCTGGGGAGGGCCACCTTGTTGTTGACCACCACCCTGAC |
| EDR875 | ScrORD2 | GTTATCAAGCTTACAATGCTCAAGCCAATTACCAGCAGCAAGGCGGTCAACAGGGTCAAGCCCAACAGCAACAACAACCTCCCTACGGTCAATCTGG |
| EDR876 | ScrORD2 | CTCCCTACGGTCAATCTGGGCCTAATCAACAAGGTCAGGGCTACTATTACAATTACTACGGTGCAGGAGACCAGTTCCCTTATAATTATGGG |
| EDR877 | ScrORD2 | GTTGAAGTTTTTGTAATTTCCACGGCCTGGTTGATAACCGTATTGGCCATTATACCCATAATTATAAGGGAACTGGTCTC |
| EDR878 | ScrORD3 | GTTATCAAGCTTACAATGCTCAAGCCGGAGGTCAAGGTCAACAGTATAATTACCAAGGTCCTCCAAATTACTATGGTCAGCAATATCAACAGCC |
| EDR879 | ScrORD3 | CTATGGTCAGCAATATCAACAGCCTTACGGCGACAATCAAGGTAATCAAAATGGCTCTGGTTTCCAGTACCCCCAAGCAGGACAAGGTCAACCTTAC |
| EDR880 | ScrORD3 | GTTGAAGTTTTTGTAATTTCCACGGCCCCCGTAGTATTGTTGCTGATAGTAGGCCTGGTAAGGTTGACCTTGTCCTGC |
| EDR881 | Scr½ORD1 | CAACAAGGTGGCTATCAACAGTACAATGGTGGCCAGCAGGCCTACGGAGGCCAACAAAATTACTTCCCTAATCCCG |
| EDR882 | Scr½ORD1 | GTTGAAGTTTTTGTAATTTCCACGGCCATAGTCTTGTTGTTGCTGTGGATAACCAGGATAATTCTGCTGTTGACCGGGATTAGGGAAGTAATTTTGTTGG |
| EDR883 | Scr½ORD2 | CAACAAGGTGGCTATCAACAGTACAATGGCTATCAGCCTTATAATCAGGACTATTACCAACAAGGACAGCAAGGC |
| EDR884 | Scr½ORD2 | GTTGAAGTTTTTGTAATTTCCACGGCCCTGACCATTACCTTGTGGATTACCAGGTTGGTAGAATTGCTGGGGGGCGCCTTGCTGTCCTTGTTGG |
| EDR885 | Scr½ORD3 | CAACAAGGTGGCTATCAACAGTACAATCAACCAGGCCAGGGCGGTTACTATCAGGGTCCCCAACCTGCCTACTATAACAACCAACAACAATTCCAGGGAC |
| EDR886 | Scr½ORD3 | GTTGAAGTTTTTGTAATTTCCACGGCCACCTTGATTCTGGTCCTGATAAGGTCCCTGGAATTGTTGTTGGTTG |
| EDR946 | ScrPrP1 | CAACAAGGTGGCTATCAACAGTACAATCCACATGGAGGACAGGGACAGGGACAAGGACAACATGGAGGTGGTCCTGGAGGAGGTCATGGTGGTCAGG |
| EDR890 | ScrPrP1 | GTTGAAGTTTTTGTAATTTCCACGGCCTGGTCCAGGACCCCAACCCCAATGCCAACCACCTTGTCCAGGTCCCCACCAACCCTGACCACCATGACCTC |
| EDR947 | ScrPrP2 | CAACAAGGTGGCTATCAACAGTACAATGGTGGAGGTCCAGGAGGTTGGCATCCACCTCATCAATGGGGTGGAGGTCATCATGG |
| EDR948 | ScrPrP2 | GGGTGGAGGTCATCATGGAGGAGGTGGAGGTGGAGGTCAGCAGGGTGGTCAATGG |
| EDR893 | ScrPrP2 | GTTGAAGTTTTTGTAATTTCCACGGCCTCCTTGAGGCCATCCCCACTGACCTCCAGGCCATTGACCACCCTGCTG |
| EDR949 | ScrPrP3 | CAAGGTGGCTATCAACAGTACAATCATCCACAATGGCCACAGCAGTGGGGTGGAGGTCCTCAACCTGGAGGTCATGGTGGAGGAGGTGGACAAGGACC |
| EDR895 | ScrPrP3 | GTTGAAGTTTTTGTAATTTCCACGGCCTCCACCTTGACCTCCCCAATGACCCCAATGACCCCATCCACCTCCACCAGGTCCTTGTCCACCTCC |
| EDR1025 | ScrNuc1 | GTTATACTGCTGTTGTTGGTAGGCAGCGTAGCTGCCACCGTTCTGGTTCATGTAATCATTTTGGTTTCTTTGATTGTACTGTTGATAGCCACCTTGTTG |
| EDR1026 | ScrNuc1 | GCTGCCTACCAACAACAGCAGTATAACAATAACCAATCACAGAACTATGGTAACGCTCAACAAGGTTCGCAATTCAACTACAACAACAATCTGCAGGG |
| EDR1027 | ScrNuc-1, -3 | TTCAACTACAACAACAATCTGCAGGGATATCAAGCTGGTTTCCAACCACAG |
| EDR1030 | ScrNuc2 | GGGCATATTGGCTGCCTGAGTTATTTTGTTGCTGTTGGTAACCTCTTTGAGCGTTATCGTTTTGTTGATTGTTATTGTACTGTTGATAGCCACCTTGTTG |
| EDR1031 | ScrNuc2 | CAGGCAGCCAATATGCCCAGTACGCTATGCAGCAACAAAACGGTTACAACAACTCGTACGGTTTCAACTACAATAACAATTTGCAAGGATATCAAGCTGG |
| EDR1034 | ScrNuc3 | GGCTTGTTGCGATTGTTGACCTGAAGCGTAATAACCGTTGTTTTGTTGTTGCTGGTATCTTTGGTAATTGTACTGTTGATAGCCACCTTGTTG |
| EDR1035 | ScrNuc3 | GGTCAACAATCGCAACAAGCCAACAACAACGATATGAACCAGTACGGCAATAGCCAGCAAGGTAATGCTAACTTCAACTACAACAACAATCTGCAGGG |
| EDR236 | Common Primer | GAGACAAGCTTCAAAGTCTTCTTTGGTTTGGGAGCGGCCTGCTTTTGTTGCTTTTGAAAGTCGTTCAAAGACATACCTTGAGACTGTGGTTGGAAACCAG |
| EDR243 | Common Primer | GATTCGGCAGGTTTTGTGCCAACCTTCTTGGTAGCATTGGCCAACTTGATACCGGAACTGGAGACAAGCTTCAAAGTCTTCTTTG |
| EDR257 | Common Primer | GACCATGATTACGCCAAGCTC |
| EDR259 | Common Primer | CCAAAGCTCCCATTGCTTCTG |
| EDR261 | Common Primer | GTTCTTCGACCTTTGTTGGCTC |
| EDR262 | Common Primer | GCATCAGCACTGGTAACATTGG |
| EDR313 | Common Primer | GGCTTGAGCATTGTAAGCTTGATAAC |
| EDR322 | Common Primer | gctggtttccaaccacagtctc |
| EDR348 | Common Primer | TTCTTCATCGACTTGCTCGGAATAACATCTATATCTGCCCACTAGCAACAATGTCG |
| EDR869 | Common Primer | TTCTTCATCGACTTGCTCGG |
| EDR870 | Common Primer | ATTGTACTGTTGATAGCCACCTTGTTG |
| EDR871 | Common Primer | GATTCGGCAGGTTTTGTGCC |
| EDR896 | Common Primer | GGCCGTGGAAATTACAAAAACTTCAACTACAATAACAATTTGCAAGGATATCAAGCTGGTTTCCAACCACAGTCTC |
| EDR897 | Common Primer | GGCTTGAGCATTGTAAGCTTGATAAC |
| EDR969 | Common Primer | cgatgctactgcagtttaCATATCGTTAACAACTTCGTCATCCAC |
| EDR1008 | Common Primer | gagctactggatccacaATGTCGGATTCAAACCAAGGCAAC |
