# Supplementary material for: [PSI+] Maintenance Is Dependent on the Composition, Not Primary Sequence, of the Oligopeptide Repeat Domain
Source: PLoS One. 2011 Jul 8;6(7):e21953. doi: 10.1371/journal.pone.0021953 (PMC3132755; doi:10.1371/journal.pone.0021953)
Supplement: Text S1 — Supplemental information – Protein sequences. (DOC) [file pone.0021953.s002.doc]

**Text S1: Supplemental information – Protein sequences**

Below are the prion domain sequences for each of the constructs used in this study. In each case, the indicated amino acids were fused to amino acids 115-685 of Sup35.

Wild-type Sup35:

MSDSNQGNNQQNYQQYSQNGNQQQGNNRYQGYQAYNAQAQPAGGYYQNYQGYSGYQQGGYQQYNPDAGYQQQYNPQGGYQQYNPQGGYQQQFNPQGGRGNYKNFNYNNNLQGYQ

FP21N:

MSDSNQGNNQQNYQQYSQNGNQQQGNNRYQGYQAYNAQASQYNSYNGQQQQFQNNQYQGNRDQQGYSGYQGDAYNQQGQYYPQGNGPNYYNYGNQGYKNQQAGQQGGYANNGFN

FP21C:

MSDSNQGNNQQNYQQYSQNGNQQQGNNRYQGYQAYNAQAGQYYPQGNGPNYYNYGNQGYKNQQAGQQGGYANNGFNGLNYRYGPQYQGYYNYNQGQPPQNAYQAQQQGQQNQSN

FP24N:

MSDSNQGNNQQNYQQYSQNGNQQQGNNRYQGYQAYNAQASNNQNGQPNNPYRQNGQQYYYNPQNSYGANGQQNRYGYYQNQYYQQAGGSNQFGNQGYDKNGYQYQFQQAQYQNQ

FP24C:

MSDSNQGNNQQNYQQYSQNGNQQQGNNRYQGYQAYNAQAYQNQYYQQAGGSNQFGNQGYDKNGYQYQFQQAQYQNQLYQGYANNGNSGQQNAGNPYGGGGYQDQQQGQQYYQQP

FP26N:

MSDSNQGNNQQNYQQYSQNGNQQQGNNRYQGYQAYNAQASYQYQNNYGNQGNQGQQYNNYSQGGAQPQLQRYQYQQPYPQYYYGNQNQQYYQQYQPQQYGGANYRGQNQNFYGN

FP26C:

MSDSNQGNNQQNYQQYSQNGNQQQGNNRYQGYQAYNAQAYPQYYYGNQNQQYYQQYQPQQYGGANYRGQNQNFYGNDQYAGQYGGGNNNNGSGDQGNSNAKPQGQQNGQFNQYA

FP27N:

MSDSNQGNNQQNYQQYSQNGNQQQGNNRYQGYQAYNAQASYQGYQNGQYYQNKFYDQNGSYQGSGYNNNQYGQQQQYNYQQAGGYNYANQYGNPNQRPGQGNGQQSQQQQYQRP

FP27C:

MSDSNQGNNQQNYQQYSQNGNQQQGNNRYQGYQAYNAQANYQQAGGYNYANQYGNPNQRPGQGNGQQSQQQQYQRPQYNQYYQAQNNQPQGFQNDGYNQGGGNANGAPNYGLYQ

Scr½ORD1:

MSDSNQGNNQQNYQQYSQNGNQQQGNNRYQGYQAYNAQAQPAGGYYQNYQGYSGYQQGGYQQYNGGQQAYGGQQNYFPNPGQQQNYPGYPQQQQDYGRGNYKNFNYNNNLQGYQ

Scr½ORD2:

MSDSNQGNNQQNYQQYSQNGNQQQGNNRYQGYQAYNAQAQPAGGYYQNYQGYSGYQQGGYQQYNGYQPYNQDYYQQGQQGAPQQFYQPGNPQGNGQGRGNYKNFNYNNNLQGYQ

Scr½ORD3:

MSDSNQGNNQQNYQQYSQNGNQQQGNNRYQGYQAYNAQAQPAGGYYQNYQGYSGYQQGGYQQYNQPGQGGYYQGPQPAYYNNQQQFQGPYQDQNQGGRGNYKNFNYNNNLQGYQ

ScrORD-1:

MSDSNQGNNQQNYQQYSQNGNQQQGNNRYQGYQAYNAQAAFQQQPYGQNYPYGYSYAQQGQYQYGYNQDYYNNPYGYGQGGGQQQGGPPQQGNQQQGRGNYKNFNYNNNLQGYQ

ScrORD-2:

MSDSNQGNNQQNYQQYSQNGNQQQGNNRYQGYQAYNAQANYQQQGGQQGQAQQQQQPPYGQSGPNQQGQGYYYNYYGAGDQFPYNYGYNGQYGYQPGRGNYKNFNYNNNLQGYQ

ScrORD-3:

MSDSNQGNNQQNYQQYSQNGNQQQGNNRYQGYQAYNAQAGGQGQQYNYQGPPNYYGQQYQQPYGDNQGNQNGSGFQYPQAGQGQPYQAYYQQQYYGGRGNYKNFNYNNNLQGYQ

ScrPrP-1:

MSDSNQGNNQQNYQQYSQNGNQQQGNNRYQGYQAYNAQAQPAGGYYQNYQGYSGYQQGGYQQYNPHGGQGQGQGQHGGGPGGGHGGQGWWGPGQGGWHWGWGPGPGRGNYKNFNYNNNLQGYQ

ScrPrP-2:

MSDSNQGNNQQNYQQYSQNGNQQQGNNRYQGYQAYNAQAQPAGGYYQNYQGYSGYQQGGYQQYNGGGPGGWHPPHQWGGGHHGGGGGGGQQGGQWPGGQWGWPQGGRGNYKNFNYNNNLQGYQ

ScrPrP-3:

MSDSNQGNNQQNYQQYSQNGNQQQGNNRYQGYQAYNAQAQPAGGYYQNYQGYSGYQQGGYQQYNHPQWPQQWGGGPQPGGHGGGGGQGPGGGGWGHWGHWGGQGGGRGNYKNFNYNNNLQGYQ

ScrNuc1:

MSDSNQGNNQQNYQQYSQNGNQQQGNNRYQGYQAYNAQAQPAGGYYQNYQGYSGYQQGGYQQYNQRNQNDYMNQNGGSYAAYQQQQYNNNQSQNYGNAQQGSQFNYNNNLQGYQ

ScrNuc2:

MSDSNQGNNQQNYQQYSQNGNQQQGNNRYQGYQAYNAQAQPAGGYYQNYQGYSGYQQGGYQQYNNNQQNDNAQRGYQQQQNNSGSQYAQYAMQQQNGYNNSYGFNYNNNLQGYQ

ScrNuc3:

MSDSNQGNNQQNYQQYSQNGNQQQGNNRYQGYQAYNAQAQPAGGYYQNYQGYSGYQQGGYQQYNYQRYQQQQNNGYYASGQQSQQANNNDMNQYGNSQQGNANFNYNNNLQGYQ
